# Single‐Cell Transcriptomics Reveals Longevity Immune Remodeling Features Shared by Centenarians and Their Offspring

**DOI:** 10.1002/advs.202204849

**Published:** 2022-11-10

**Authors:** Chen Dong, Ya‐ru Miao, Rui Zhao, Mei Yang, An‐yuan Guo, Zhong‐hui Xue, Teng Li, Qiong Zhang, Yanfeng Bao, Chen Shen, Chi Sun, Ying Yang, Xi‐xi Gu, Yi Jin, Rong Li, Min Xu, Jia‐xin Guo, Zhi‐ying Zong, Wei Zhou, Mei He, Dan‐ni Wang, Jian‐you Su, Xiao‐ming Zhang, Xu‐hui Zeng, Jian‐lin Gao, Zhi‐feng Gu

**Affiliations:** ^1^ Research Center of Clinical Medicine Research Center of Gerontology and Longevity Key Laboratory of Immunology Research Center of Nursing Department of Rheumatology Affiliated Hospital of Nantong University Nantong University Nantong 226001 China; ^2^ Center for Artificial Intelligence Biology Hubei Bioinformatics & Molecular Imaging Key Laboratory Key Laboratory of Molecular Biophysics of the Ministry of Education College of Life Science and Technology Huazhong University of Science and Technology Wuhan 430074 China; ^3^ Key Laboratory of Molecular Virology & Immunology Institut Pasteur of Shanghai Chinese Academy of Sciences Shanghai 200025 China; ^4^ Department of Geriatrics Affiliated Hospital of Nantong University Nantong University Nantong 226001 China; ^5^ Laboratory Center Affiliated Hospital of Nantong University Nantong University Nantong 226001 China; ^6^ Institute of Reproductive Medicine Medical School Nantong University Nantong 226001 China

**Keywords:** centenarian, immune remodeling, longevity, single‐cell transcriptome, T cell

## Abstract

Centenarians, who show mild infections and low incidence of tumors, are the optimal model to investigate healthy aging. However, longevity related immune characteristics has not been fully revealed largely due to lack of appropriate controls. In this study, single‐cell transcriptomic analysis of peripheral blood mononuclear cells (PBMCs) derived from seven centenarians (CEN), six centenarians’ offspring (CO), and nine offspring spouses or neighbors (Control, age‐matched to CO) are performed to investigate the shared immune features between CEN and CO. The results indicate that among all 12 T cell clusters, the cytotoxic‐phenotype‐clusters (CPC) and the naïve‐phenotype‐clusters (NPC) significantly change between CEN and ontrol. Compared to Control, both CEN and CO are characterized by depleted NPC and increased CPC, which is dominated by CD8^+^ T cells. Furthermore, CPC from CEN and CO share enhanced signaling pathways and transcriptional factors associated with immune response, and possesse similar T‐cell‐receptor features, such as high clonal expansion. Interestingly, rather than a significant increase in GZMK^+^ CD8 cells during aging, centenarians show accumulation of GZMB^+^ and CMC1^+^ CD8 T cells. Collectively, this study unveils an immune remodeling pattern reflected by both quantitative increase and functional reinforcement of cytotoxic T cells which are essential for healthy aging.

## Graphical abstract

Immune remodeling patterns of quantitative increase and functional reinforcement of cytotoxic T‐cells in centenarian and their offspring, assessed by single‐cell RNA sequencing, fluorescence activated cell sorting and T cell receptor sequencing, provides insight into successful interventions against aging and exposes the path for healthy aging.

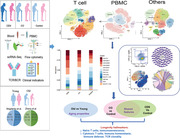

## 1 Introduction

The medical and social service burdens resulting from an aging society worldwide require clarification of the mechanisms underlying longevity. Longevity is a natural complex phenomenon characterized by an increased healthy lifespan with delay or absence of age‐related diseases and influenced by many factors including genetics, environment, culture, and psychosocial status.^[^ ^1^ ^]^ All of these factors can impact the immune system, which is functionally enhanced in long‐lived individuals as shown in previous transcriptomic and proteomic analysis.^[^ ^2^ ^]^ Therefore, elucidating the kinetic process and characteristics of the immune system during healthy aging may clarifying the mechanisms underlying longevity. Centenarians are often considered as the representative of extreme human longevity. The lives of centenarians are usually accompanied by mild infections and low tumor incidence, often a result of uncommonly prolonged robust immunity.^[^ ^3^ ^]^ Hence centenarians are an optimal model for understanding the immune signature of healthy aging.

Immunosenescence is incontrovertible a major challenge in the elderly population.^[^ ^4^ ^]^ The clinical features of immunosenescence point to dysfunction in innate immunity with the increased production of proinflammatory cytokines by macrophages and fibroblasts, which drive most age‐associated diseases, such as diabetes and atherosclerosis.^[^ ^5^ ^]^ Nevertheless, the immune system of centenarians is more likely to maintain appropriate immunity through aging.^[^ ^6^ ^]^ Compared to the general elderly population, decline of natural killer (NK) cell activity, lymphocytes proliferation, and functional T cell repertoires are relatively moderate in centenarians.^[^ ^7^ ^]^ Most recently, a single cell transcriptomic study reported a marked increase in cytotoxic CD4^+^ T cells with massive clonal expansion in circulating lymphocytes as a signature of Japanese supercentenarians.^[^ ^8^ ^]^ This study highlighted the potential of single cell sequencing to characterize the complex immune system in centenarians, these are commonly flawed by the lack of appropriate controls.

To circumvent the lack of appropriate age‐matched controls for centenarians, researchers recruited centenarians’ offspring into consideration.^[^ ^9^ ^]^ When compared with age‐matched controls without centenarian parents, centenarian offspring showed better health and less frail, suggesting relatively longer lifespan.^[^ ^10^ ^]^ In addition, they showed well‐preserved immunological profile and marked reduced risk for CVD, hypertension, diabetes, stroke, myocardial infarction, Alzheimer's disease, dementia, and reduced mortality (81%) than controls.^[^ ^9^, ^11^ ^]^ Consistently, centenarian offspring exhibited 4‐ and 17‐fold higher odd ratio for longevity.^[^ ^9a^ ^]^ Furthermore, longevity is a phenotype significantly affected by inheritance, with a protective genetic contribution of more than 33% in males and 48% in females.^[^ ^12^ ^]^ Consistently, immune cell composition is partly determined by genetic factors.^[^ ^13^ ^]^ Therefore, it is logical that, compared to age‐matched controls, centenarian offspring may share their parents immune characteristic and transcriptomic signature important for longevity.

In this study, we collected peripheral blood mononuclear cells (PBMC) of centenarians (CEN), centenarians’ offspring (CO), and offspring's spouses and elderly neighbors whose parents died between 63 and 77 years old in the same village (Control, age‐matched to CO) from Rugao, a blue zone in China.^[^ ^14^ ^]^ Single‐cell RNA sequencing, T cell receptor (TCR) sequencing, and flow cytometry analysis were performed to explore longevity related global immune features and functional mechanism, especially immune characteristics shared between CEN and CO.

## 2 Results

### 2.1 scRNA‐seq Reveals Cellular Profiling of Peripheral Immune Cells

**Figure 1:**
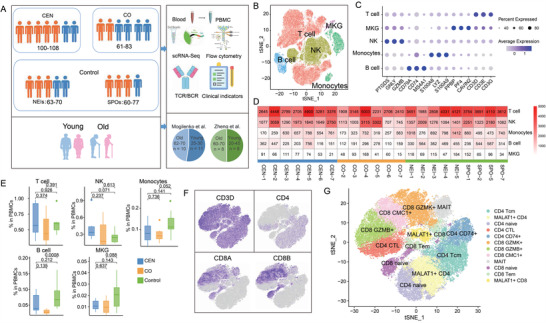
Single‐cell transcriptome profiled PBMCs in all samples. A) Project landscape. PBMCs of 7 centenarians (CEN), 6 centenarian offsprings (CO), 4 spouses of offspring (SPO), and 5 neighbors of offsprings (NEI) were collected and sequenced. Samples from SPO and NEI groups were taken as Control group. Besides, two cohorts of young and aged adults were collected from published work. B) 2D tSNE visualization of cell clusters from PBMCs of all samples. MKG, the abbreviation of megakaryocyte. C) Expression of top three DEGs per cell type. The size of the dot means proportion of cells with expression in each cluster. Color of the dot means average expression of all cells in each cluster. D) Heatmap depicting the number of cells identified per participant. E) Box plot showing the abundance of major cell types in CEN, CO, and Control groups, *p*‐value was estimated by One‐side Wilcoxon rank sum test. F) Expression of marker genes for T cells (CD3D), CD4 T cells (CD4), and CD8 T cells (CD8A, CD8B) in all samples. G) 2D tSNE visualization of T cell clusters in all samples.

Participants were 100–108 (101.71 ± 3.09), 61–83 (70.50 ± 9.73), and 60–77 (67.20 ± 3.03) years old for CEN, CO, and Control groups, respectively, without any clear clinical indicators of disease (Figure S1, Supporting Information). PBMCs were collected and processed separately for single‐cell RNA sequencing, TCR sequencing, and flow cytometry. Data analysis regarding general aging was performed as previously published by Zheng et al.^[^ ^15^ ^]^ and Mogilenko et al.^[^ ^16^ ^]^ (**Figure** **1**A). After quality control, the total numbers of the recovered cells were 4345–8358 in the CEN group (median 4653), 3354–9314 in the CO group (median 4324), and 4903–7573 in the Control group (median 6274) (Figure S2A, Supporting Information). The median of estimated genes per cell was 1163–1579 (Figure S2B, Supporting Information).

Based on specifically upregulated gene signatures in each cluster, 5 major cell types were identified: B cells (*CD79A*, *CD74*, *MS4A1*), monocytes (*S100A8*, *S100A9*, *LYZ*), NK cells (*PTGDS*, *GNLY*, *GZMB*), progenitors of megakaryocytes (Pro‐MKG, *PPBP*, *PF4*, *CAVIN2*), and T cells (*CD3D/E/G*) (Figure 1B–D). Compared to Control individuals, there was no significant change in the proportion of above cell types in CEN group (Figure 1E). T cells, have been reported to vary and play crucial roles in immunosenescence during aging.^[^ ^15^, ^17^ ^]^ These accounted for the highest proportion in PBMCs (Figure 1D,E) and were defined as CD4 T and CD8 T cells based on the expression of CD4 and CD8A/B (Figure 1F). To investigate longevity‐associated changes in immune cell subtypes of CD4 T and CD8 T, T cells were further clustered into 12 distinct subtypes based on distinct upregulated gene signatures in each cluster (Figure 1G; and Figure S3, Supporting Information).

### 2.2 Immune Remodeling of CEN in Naïve and Cytotoxic T‐Cells

**Figure 2:**
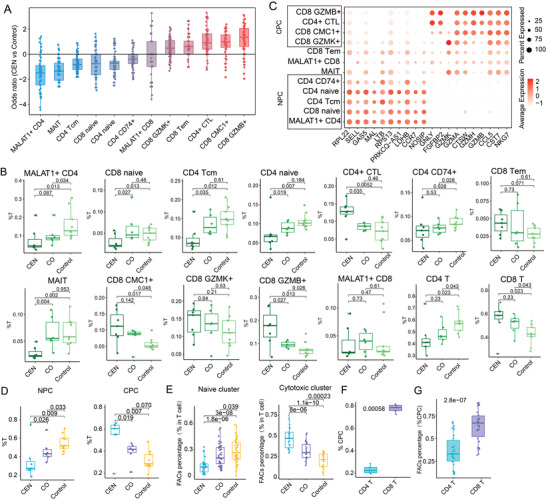
Characteristic of enriched or depleted T cell subtypes in CEN and CO. A) Enrichment of cells in CEN or Control groups is calculated as the log transformed odds ratio of cell in each group. Positive value means enriched in CEN, negative value means enriched in Control. B) Box plot showing the percentages of T cell subtypes in total T cells. *p*‐value was estimated by One‐side Wilcoxon rank sum test in this and all subsequent figures unless otherwise indicated. C) Dot plot of average expression of signature genes in NPC and CPC. D,E) Box plot showing the percentages of CPC and NPC in total T cells calculated from scRNA‐seq result D) and Fluorescence activated Cell Sorting (FACs) E). F,G) Box plot showing the percentages of CD4 and CD8 T cell in total CPC cells calculated from scRNA‐seq result F) and FACs G).

Enrichment of cell cluster in samples from different groups was computed as the log odds ratio between the frequency of each subset in CEN versus Control, across all pairs. The results showed GZMB^+^ CD8 T cells, CMC1^+^ CD8 T cells, and CD4^+^ CTL subsets were enriched in CEN (median log2 odds ratio: 1.344, 0.984, 0.9, respectively). Inversely, T cell subsets such as CD4 naïve, CD8 naïve, and central memory CD4 (CD4 Tcm) were enriched in Control (**Figure** **2**A). Statistical analysis showed the abundance of these cell subsets were significantly different between CEN and Control samples (*p* value < 0.05). Interestingly, CO individuals exhibited the same tendency as CEN compared to the age‐matched Control samples (Figure 2B). Subsequently, we investigated shared characteristics among cell subsets significantly enriched or depleted in CEN. The results showed that cell subsets enriched in CEN highly expressed genes with cytotoxic function (such as *GZMA, GZMH, GZMB*) (Figure 2C),^[^ ^18^ ^]^ and were therefore defined as cytotoxic phenotype clusters (CPC). Contrastingly, cell subsets enriched in Control individuals highly expressed naïve markers such as *CCR7*, *SELL*, and *NOSIP*, and were denoted as naïve phenotype clusters (NPC) (Figure 2C). When comparing the proportions of CPC and NPC as a whole among these three groups, it is obvious that CPC were significantly increased, while NPC were substantially decreased in both CEN and CO compared to Control (Figure 2D). Furthermore, the proportion of CPC and NPC was confirmed by flow cytometry based on larger sample cohort (Figure 2E, *n* = 30 for each group). Worthy of note, within CPC, CD8 positive clusters represented the majority of cytotoxic T cells in CEN, both by single‐cell transcriptomics (Figure 2F) and flow cytometry analysis (Figure 2G). Considering CO were compared to the age‐matched Controls, these results suggest the increase of CPC and decrease of NPC are a heritable hallmark of healthy aging.

### 2.3 T Cells Exhibited Specific Infiltration Features Related to Longevity and Aging

**Figure 3:**
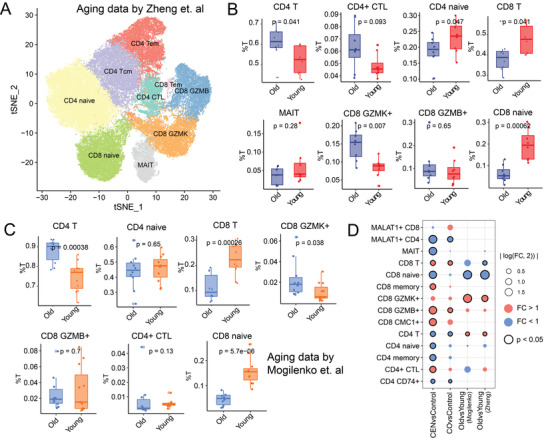
Characteristics in immune cell abundance distribution of longevity and aging. A) 2D tSNE visualization of cell clusters from PBMCs of young and aged samples by Zheng et al. B,C) Box plot showing the percentages of T cell subtypes in total T cells in the cohorts by Zheng et al. B) and Mogilenko et al. C), separately. D) Dot plot showing significantly changed immune cells during aging and longevity in our longevity cohort and aging cohorts by Zheng et al and Mogilenko et al. Size of the dot represents the absolute value of log2‐transformed fold change between CEN versus Control/CO versus Control/Aging versus Young. Red color means fold change larger than 1, blue color means fold change smaller than 1. “x” indicates the corresponding cell type was absent in the cohort. Dot with black circle means there is significantly different between groups in immune cell distribution.

CEN individuals were naturally older than Controls, so we wondered which immune factors may be associated with healthy aging. For this purpose, we investigated the difference between longevity and aging in T cell infiltration using previously published data. Zheng et al.^[^ ^15^ ^]^ (*n* = 16, 8 old, 8 young) described a total of 9 T cell subset clusters identified in elderly subjects, 8 of them were also identified in this study (**Figure** **3**A). Additionally, we downloaded annotated cell barcode data published by Mogilenko et al.^[^ ^16^ ^]^ (*n* = 21, 10 old, 11 young). Statistical analysis showed significant differences in T cell infiltration shared between samples from age and young donors in cohorts by Zheng et al. and Mogilenko et al. (Figure 3B,C). For example, accumulation of total CD4 T cell and GZMK^+^ CD8 T cell, and a decrease of total CD8 T cell and CD8 naïve T cell in the elderly group. Therefore, changes in CD4 and CD8 T cells during aging in general elderly population seems opposite to those in longevity population (CEN) (Figure 2B).

Combined with the changes in T cell infiltration described in the former section, we observed that T cells exhibited specific infiltration features related to longevity and aging. For the subsets of NPC clusters, CD4 naïve T cells were significantly decreased in the CEN versus Control comparison but not in Old versus Young comparison. Besides, the abundance of MALAT1^+^ CD4 and CD74^+^ CD4 T cells were also significant decreased in CEN. However, the change of MALAT1^+^ and CD74^+^ CD4 T cells during aging was unclear, as these cell types were not identified in both aging related cohorts. Nevertheless, CD8 naïve T cells showed consistent changes during aging and longevity (Figure 3D). Consistent with the work by Hashimoto et al.,^[^ ^8^ ^]^ we observed a significant increase of CD4^+^ CTL in centenarians (Figure 2B), which showed no significant change during aging (Figure 3B–D). Interestingly, among three CD8 subsets with cytotoxic function, CD8 GZMK^+^ cells were significantly accumulated in old group but not in CEN (comparing to Control group) (Figure 3D). Meanwhile, CEN samples showed significantly increased GZMB^+^ and CMC1^+^ CD8 T cells, but GZMB^+^ CD8 T cells were failed to be observed in elderly and young individuals in previous studies (Figure 3D). Except for CD4^+^ CTL, specific T‐cell subset associated to longevity were also identified in CO compared to age‐matched Control (Figure 3D).

### 2.4 CPC of CEN and CO were More Powerful in Immune Homeostasis Process

**Figure 4:**
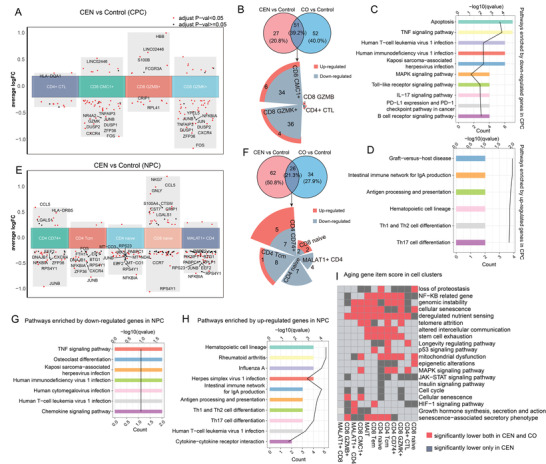
Differently expressed genes (DEGs) and enriched pathways of CPC and NPC cell clusters in the comparison of CEN versus Control and CO versus Control. A) Differential gene expression analysis showing up‐ and down‐regulated genes across CPC cell clusters. An adjusted *p*‐value smaller than 0.05 is indicated in red, while an adjusted *p*‐value larger than 0.05 is indicated in black. B) Venn plot showing the shared DEGs between CEN versus Control and CO versus Control comparisons. Sunburst plot showing the detailed number of DEGs in each cell cluster of CPC. The size of the red and gray areas represents the number of shared up‐regulated and down‐genes between CEN and CO, respectively. C,D) Bar plot showing KEGG pathways enriched by down‐regulated C) and up‐regulated D) genes shared by CEN and CO in CPC cell clusters. E) Differential gene expression analysis showing up‐ and down‐regulated genes across NPC cell clusters. F) Venn plot showing the shared DEGs between CEN versus Control and CO versus Control comparisons. Sunburst plot showing the detailed number of DEGs in each cell cluster of NPC. G,H) Bar plot showing KEGG pathways enriched by down‐regulated G) and up‐regulated H) genes shared by CEN and CO in NPC cell clusters. I) Heatmap showing the difference of aging score between CEN versus Control and CO versus Control in each cell cluster.

Having shown significant changes in the proportion of CPC and NPC in CEN, we next focused on the cell‐subtype specific gene signatures associated with longevity. We performed differential expression analysis in CPC or NPC cell subsets between CEN versus Control and CO versus Control, separately. For CPC subset cluster, differentially expressed genes (DEGs) analysis showed CMC1^+^ CD8 T cell had the most significant longevity‐related changes, followed by GZMK^+^ CD8 T cell (**Figure** **4**A). Moreover, there were shared DEGs between both GZMK^+^ and CMC1^+^ CD8 T cell clusters, such as JUNB, ZFP36, FOS, and CXCR4. Combining the results of CEN versus Control and CO versus Control, we found that almost 40% DEGs were shared by CEN and CO, such as down‐regulated FOS, JUNB, NR4A2, and CXCR4 (Figure 4B; and Table S1, Supporting Information). These were mainly related to aging, inflammation, immune escape, and T cell dysfunction.^[^ ^19^ ^]^ Contrastingly, CEN and CO showed increased expression of HLA‐DQA1, GIMAP4, PLEK, and CTSW (Table S1, Supporting Information), associated with enhancement of immune cell differentiation and activation.^[^ ^20^ ^]^

To further explore the biological implications of longevity‐related DEGs, we performed pathway enrichment analysis of dysregulated genes in CEN and CO. Shared down‐regulated genes by CEN and CO were mainly enriched in apoptosis, TNF signaling pathway and various virus infection pathway (Figure 4C). These were enriched by most cell subsets in CPC (Figure S4A, Supporting Information). In addition, shared up‐regulated genes by CEN and CO were mainly enriched in the pathways related to T cell functional homeostasis, including Th1 and Th2 cell differentiation, Th17 cell differentiation and antigen processing and presentation (Figure 4D), also enriched by most cell subsets in CPC (Figure S4B, Supporting Information). Among NPC clusters, CD74^+^ CD4 T and CD4 Tcm were most associated with longevity based on the number of DEGs (Figure 4E,F). Moreover, shared down‐regulated genes by CEN and CO were enriched in TNF signaling pathway, Osteoclast differentiation and chemokine signaling pathway (Figure 4G). Similar to CPC clusters, shared up‐regulated genes by CEN and CO in the NPC clusters were enriched in pathways related to immune response process, such as hematopoietic cell lineage, Intestinal immune network for IgA production, and Antigen processing and presentation (Figure 4H). Together, these findings indicate the ability to reverse immunosenescence and inflammation, and enhancement of immune defense and homeostasis may drive healthy aging in long‐lived individuals (centenarians).

We subsequently calculated the aging score of samples in CEN, CO, and Control based on the aging‐related gene sets collected from Aging Atlas database.^[^ ^21^ ^]^ Statistical analysis showed many aging related signaling pathways were significantly lower in CEN and CO, including NF‐KB related gene, genomic instability, cellular senescence, deregulated nutrient sensing, telomere attrition, altered intercellular communication, stem cell exhaustion (Figure 4I). These data further support that T cells, especially NPC cells, are “younger” in CEN and CO, and contribute to longevity.

### 2.5 CEN and CO Shared Transcriptional Factors for Immune Homeostasis

**Figure 5:**
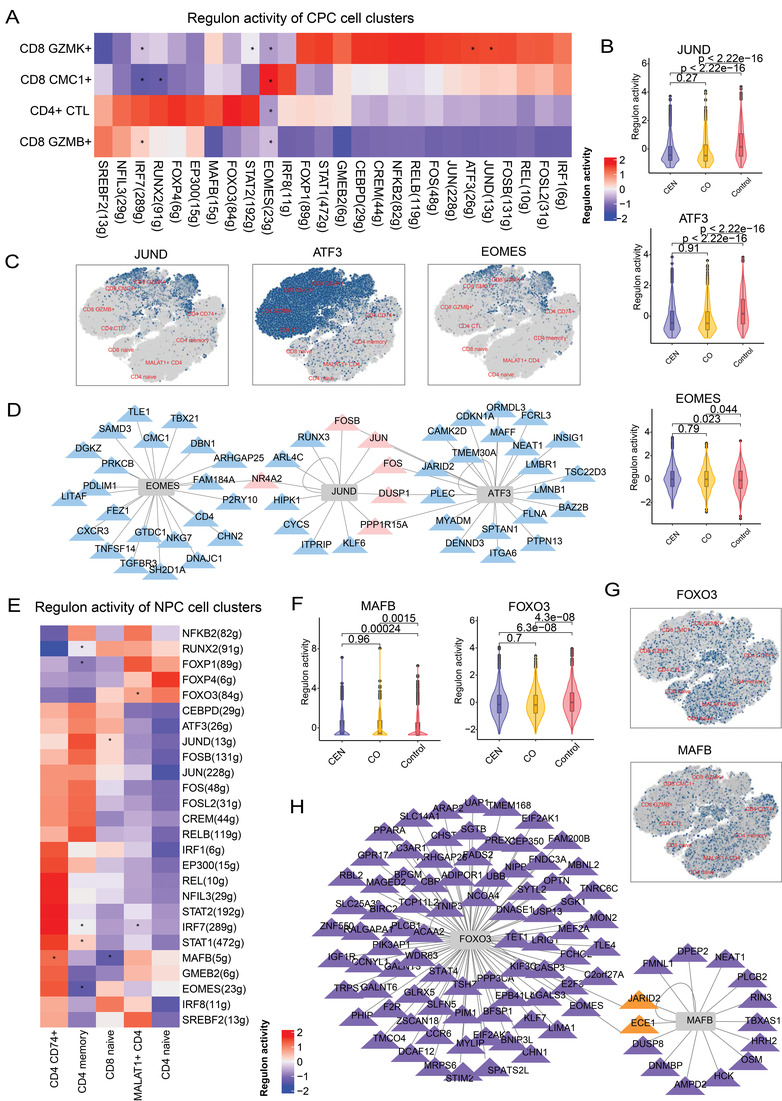
The regulatory characteristics of longevity. A) Heatmap showing regulon activity of CPC cell clusters in CEN. The number in the name of y‐axis represents genes targeted by the corresponding TF. “*” means shared TFs with the regulon activity significantly different both in the CEN versus Control and CO versus Control comparison, which is defined as longevity‐related TF. B) Box plot showing the regulon activity of longevity‐related TFs in CEN, CO, and Control groups, *p*‐value was estimated by Wilcoxon rank sum test. C) 2D tSNE visualization of regulon activity of longevity‐related TFs in T cell clusters. D) TF‐gene regulatory network for longevity‐related TFs, TFs are indicated in rectangles, and genes are indicated in triangles. Genes with blue color are targeted by one TF, and genes with pink color are targeted by two TFs. E) Heatmap showing regulon activity of NPC cell clusters. The number in the name of *y*‐axis represents genes targeted by the corresponding TF. “*” means shared TFs with the regulon activity significantly different both in the CEN versus Control and CO with Control comparison, which is defined as longevity‐related TF. F) Box plot showing the regulon activity of longevity‐related TFs in CEN, CO, and Control groups, *p*‐value was estimated by Wilcoxon rank sum test. G) 2D tSNE visualization of regulon activity of longevity‐related TFs in T cell clusters. H) TF‐gene regulatory network for longevity‐related TFs, TFs are indicated in rectangles, and genes are indicated in triangles. Genes with purple color are targeted by one TF, and genes with orange color are targeted by two TFs.

As the regulation of T cell differentiation and function is largely coordinated by the activity of transcriptional factors (TFs),^[^ ^22^ ^]^ we further examined the specific active regulons, TFs and their target gene modules, in CPC or NPC clusters by SCENIC.^[^ ^23^ ^]^ A set of 26 high‐confidence regulons in CPC cell subsets were identified, which activities were calculated by AUCell function of SCENIC (**Figure** **5**A,B). CPC subsets in CEN and CO displayed significantly different activity of regulons corresponding to certain TFs (defined as longevity‐related TFs), such as *JUND*, *EOMES*, and *IRF7*. Among CPC clusters, GZMK^+^ CD8 T cell exhibited higher activity of JUND and ATF3 regulon (Figure 5A,C), which drive cellular senescence and T cell homeostasis disorder.^[^ ^24^ ^]^ Interestingly, their activities were significantly higher in Control individuals, suggesting the aging status of Control group (Figure 5B). In addition, EOMES was the most active regulon in CMC1^+^ CD8 T cell (Figure 5A,C), and EOMES activity was significantly higher in CEN and CO (Figure 5B), which can contribute to the development and differentiation of immune cells.^[^ ^25^ ^]^ Moreover, TF‐gene regulatory network analysis showed some shared target genes between these TFs (especially for JUND and ATF3), such as *FOS, JUN, FOSB, DUSP1*, and *PPP1R15A* (Figure 5D). These are closely related to T cell dysfunction, immune homeostasis, immune escape, tumor progression, and hypertension.^[^ ^19^, ^26^ ^]^

For NPC clusters, we identified 26 high‐confidence regulons with *RNUX2, FOXP1, FOXO3, JUND, IRF7, STAT1, MAFB, EOMES* showing significantly different regulon activity in CEN and CO compared to Control (Figure 5E). Among NPC clusters, CD74^+^ CD4 T cell showed higher activity of MAFB regulon (Figure 5E,G), again more active in CEN and CO (Figure 5F). MAFB was reported to alleviate inflammation by restoring the Th1/Th2/Th17 imbalance.^[^ ^27^ ^]^ On the other hand, FOXO3 had relative higher activity in MALAT1^+^ CD4 T cells (Figure 5G). This is a key regulator to translate environmental stimuli into specific gene expression.^[^ ^28^ ^]^ FOXO3 activity was significantly lower in CEN and CO than in Control (Figure 5F). Moreover, both MAFB and FOXO3 targeted JARID2 and ECE1 (Figure 5H), which play important regulatory roles in maintaining cell homeostasis and resisting Alzheimer's and other diseases.^[^ ^29^ ^]^

### 2.6 CEN and CO Shared Reinforced Clonal Expansion of TCR

**Figure 6:**
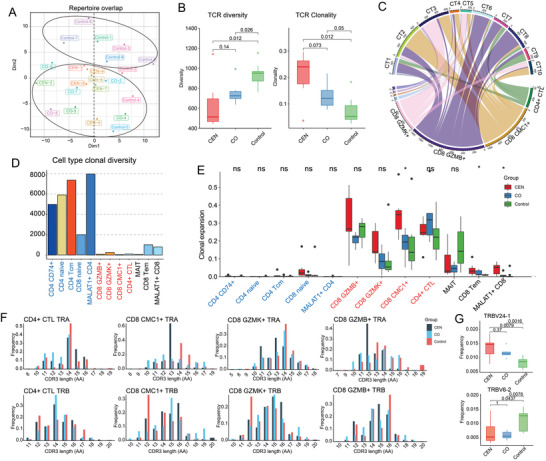
TCR profiles characteristics of longevity. A) The result of K‐means clustering analysis shows the gene usage characteristics of TCR in CEN, CO, and Control samples. B) Boxplot showing TCR diversity in 1500 T cells per sample (left) and TCR clonality of samples calculated by Shanno's Entropy. The clonality value range from 0 (polyclonal distribution) to 1 (monoclonal distribution). P is estimated by Wilcoxon rank sum test. C) Circus plot showing top10 TCR clone type (CT1‐CT10) distribution among T cell clusters. D. Barplot showing TCR diversity of each T cell cluster estimated by inverse Simpson index. Cell name with blue color means NPC cell clusters, cell name with red color means CPC cell clusters. E) Boxplot showing clonal expansion levels of T cell clusters quantified by STARTRAC‐expa for each sample, *p*‐value was estimated by Wilcoxon rank sum test. “*,” *p* < 0.05; ns, not significant. F) Bar plot showing CDR3 length distribution of TRA chain and TRB chain of cells in CPC clusters in CEN, CO, and Control groups. G) Boxplot showing the distribution of TRBV24‐1 and TRBV6‐2 are significantly different in CEN, CO, and Control groups, *p*‐value was calculated by Wilcoxon rank sum test.

The adaptive immune system's ability to respond to varieties of antigens depends on an extensive repertoire of unique TCR. With aging, the TCR diversity of naïve T cells, clonal expansion, and the richness of the entire memory T cell repertoire become limited and decrease, but elderly individuals can still possess a diverse T cell repertoire.^[^ ^30^ ^]^ We focused on longevity‐related alterations in the TCR repertoire and its overlap across all samples (**Figure** **6**A). Compared to Control individuals, the TCR diversity of CEN was notably decreased, and showed a similar decreasing trend in CO (Figure 6B). TCR clonality per sample was calculated by Shannon's Entropy described in TCRdb,^[^ ^31^ ^]^ showing that TCR clonality in CEN and CO individuals was significantly higher than Control (Figure 6B).

We next analyzed TCR clone type and observed that the top10 TCR clonotypes were distributed mostly among CPC clusters (Figure 6C) with higher clonal expansion in CPC than in NPC (Figure S5, Supporting Information). Moreover, we observed that TCR diversity was higher in NPC than CPC (Figure 6D), which was consistent with a previous report where TCR diversity was extended by homeostatic proliferated naïve T cells.^[^ ^30a^ ^]^ Subsequently, we investigated the TCR clonality of both cell clusters. TCR expansion analysis by STARTRAC showed that the clonal expansion index was predominantly higher in CPC than NPC (Figure 6E).

Diversity of TCR is generated by rearranging the V and J segments of TCR alpha (TRA) and V, D, and J segments of the TCR beta (TRB) genes. In this work, we investigated the length distributions of CDR3 sequences in CPC clusters among CEN, CO and Control groups. For example, for CD4^+^ CTL TRA, most of samples had a sequence length of 14 Amino acids (AA), while most CEN samples had a sequence length of 13 AA for CMC1^+^ CD8 T cell and 14 AA in Control (Figure 6F). Subsequently, we investigated the distribution of V and J genes in CEN, CO, and Control. The frequency of TRBV24‐1 was significantly higher both in CEN and CO compared with Control (Figure 6G; and Figure S6, Supporting Information), while TRBV6‐2 exhibited the oppositely tendency (Figure 6G), suggesting their potential role in longevity and aging.

## 3 Discussion

Aging is a natural phenomenon across the animal kingdom. Throughout humans’ lifespan, immunosenescence, including loss of CD3^+^, CD4^+^, and CD8^+^ T cell, was considered as remodeling of impaired immunoregulatory function in the elderly.^[^ ^17^, ^32^ ^]^ Centenarians are recognized as the model of healthy aging, whose immunosenescence is no longer considered as “decline” or “reduction,” but tends to be seen as “positive remodeling.”^[^ ^17a^ ^]^ In this study, we performed single‐cell transcriptomic and TCR repertoire analysis on PBMCs derived from CEN, to investigate the positive remodeling signature which may be essential for healthy aging. Most importantly, we recruited CO to understand immune heritable traits shared between CEN and their offspring potentially involved the immune remodeling pattern associated with longevity. Compared to Controls, immune remodeling in CEN is characterized by significantly increased CPC and depleted NPC, a feature shared by CO. In addition, functional changes in the immune cells of CEN and CO were also observed, represented by altered genes, pathways and transcriptional activity associated with T cell homeostasis and reverse of immunosenescence. Furthermore, both CEN and CO showed low diversity and high clonal TCR expansion. Finally, because CO and Control had similar age distribution, the immune alterations here identified should represent the positive and heritable remodeling characteristics important for longevity.

Our analysis suggested that the CPC defined here may be crucial for robust immune surveillance in CEN. The CPC may contribute to longevity through three aspects: by their increase abundance, enhancement of homeostasis, and expansion of specific clonalities. Consistent with the study performed by Hashimoto K.,^[^ ^8^ ^]^ CD4^+^ CTL was a distinguished cluster between aging and longevity (Figure 3D). Besides this significant increase in CD4^+^ CTL in supercentenarian, we also show that GZMB^+^ and CMC1^+^ CD8 T cells were increased in CEN and CO (Figure 2B). Importantly, the CD8^+^ cells accounted for 80% CPC in CEN (Figure 2F,G), suggesting their crucial role for healthy aging. Worth of note, the percentage of CD4^+^ CTL in supercentenarian (mean, 25.3% of total T cells) is higher than that revealed in our study (mean, 12.7% of total T cells), perhaps because the average age of those supercentenarian was 10 years older than the CEN cohort in this study.

The T cells’ ability to respond to external pathogens is highly dependent on the TCR repertoire diversity of naïve T cells, which is remarkably decreased in the elderly.^[^ ^33^ ^]^ In this study, TCR diversity in CEN and CO were both decreased (Figure 6B), consistent with the observed decrease of abundance of NPC cells in these two groups (Figure 2D,E). On the other hand, the clonal expansion of TCR in CEN and CO were both increased (Figure 6B), suggesting that TCR clonal expansions in CEN may not be entirely associated with aging, and that loss of TCR diversity may not always reflect weakened response to external pathogens. Clonal expansion in CEN may be targeted at specific pathogens providing better protection against infection or tumor, as reported by Hashimoto K et al.^[^ ^8^ ^]^ In addition, we observed the distribution difference of CDR3 length among CEN, CO, and Control groups (Figure 6F). Previously, CDR3 length distribution of the TCR gene families demonstrated specific utilization patterns in PBMCs,^[^ ^34^ ^]^ thus the different length distribution of TCR in CEN and CO group may associate with the immune response process during longevity.

The delay or absence of age‐related disorders occurring in CEN, and perhaps in CO, relies on their sustained immune ability to suppress tumor, anti‐infection or clearing senescent cells. As a potential longevity population, the offspring of centenarians retained more active immunological parameters and exhibited a delayed onset of age‐related diseases than the average elderly population.^[^ ^35^ ^]^ Previous studies in humans and rodents have shown that variation in circulating T cells’ levels is partially heritable, and 25–50% of interindividual variations in cellular composition and functional responses in the immune cell compartment were attributed to genetic factors.^[^ ^13^, ^36^ ^]^ In this study, we observed that CEN and CO shared specific immune remodeling features, further suggesting the contribution of genetic background to longevity. Alike to the shared immune features in this study, Xiao et al. described gene expression features of Chinese centenarian and that enhanced autophagy‐lysosomal activity could be partially passed on from centenarian to their offspring.^[^ ^37^ ^]^ However, the underlying mechanisms by which centenarian offspring “inherit” these features from their parents could be very complicated and definitely require further investigation.

During general aging, T cells in PBMCs undergo a significant remodeling process. The work by Zheng et al. showed the polarization from naive and memory cells to effector, and cytotoxic cells with age. Besides, Mogilenko et al. proved the accumulation of GZMK^+^ CD8^+^ T cells is a conserved hallmark of immune aging.^[^ ^15^, ^16^ ^]^ As this study introduces CO and their comparison with young and elderly populations, our findings may help distinguish immune remodeling differences between general aging and healthy aging. The CD4/CD8 T cell ratio changes during general aging, but contrasted in CEN (Figure 3B,C). A possible explanation would be that CD4 T cells may increase, while CD8 T cells decrease in the elderly; while CD4 T cells are depleted and CD8 T cells accumulated during healthy aging in CEN (Figure 2B). During longevity, CD4^+^ CTL cells, GZMB^+^ and CMC1^+^ cytotoxic cells were also substantially enriched (Figure 2B). However, it is the GZMK^+^ cells that show increase during general aging (Figure 3B,C). Interestingly, although there was no further increase of GZMK^+^ cells during longevity (Figure 2B), the GZMK^+^ cells in CEN and CO exhibit a status of delayed senescence compared to those in the general elderly population (Figure 3D), implying that this T cell cluster might also experience a positive remodeling and be important for successful aging. In addition to GZMK^+^ cells, the NPC during successful aging also showed a status of delayed senescence compared to those in general elderly controls (Figure 3D). Perhaps universal rejuvenation of T cell might be another distinguished feature separating healthy and general aging. Overall, our results are consistent with the idea that CEN experience an immune remodeling process inclining to the immune status of young population.^[^ ^38^ ^]^

In conclusion, we reported the shared immune remodeling pattern of centenarians and their offspring, which was represented as quantity and functional changes in the T cell compartment: the accumulation of cells with cytotoxic phenotype and depletion of cells with naïve phenotype, enhanced T cell homeostasis, and reinforced TCR clonal expansion. Taken together, these findings may provide strategies for the intervention of age‐related disorders and the promotion of healthy aging. Still, the sample of this study was limited, and future studies should validate our findings.

## 4 Experimental Section

### Experimental Design

The aim of the study was to explore the characteristic of immune system related to longevity and healthy aging. CEN is the important and ideal biological model in the scientific field of antitumor or anti‐infection and longevity or healthy aging with insufficient and ambiguous report. In addition, considering the potential impact of inheritance, environment, and culture on health, it is necessary to conduct investigations of clustered CEN and CO who share the same culture and environment in a small living area and it is committed to the research. In this study, 22 samples containing 7 CEN, 6 CO, and 9 Control (age‐matched to CO) from Rugao city, which is a blue zone in China, were collected. Single‐cell sequencing and bioinformatics analysis for PBMC and T cells of the 22 samples were performed in this study to explore the global features of immune remodeling related to longevity.

### Participants and Samples

Morning blood samples of CEN, CO, and Control groups from Rugao City were collected in tubes containing ethylene diamine tetraacetic acid (EDTA) or heparin. Demographic (gender, age) and clinical variables (complete blood counts, basic metabolic panel, liver, and renal function, blood glucose, lipid profile, and blood pressure) were performed to verify samples without any clear clinical indicators of disease. PBMCs were isolated by density centrifugation using Ficoll‐Paque PLUS within 4 h. Each blood sample was diluted with an equal volume of normal saline and centrifuged at 500 × g for 10 min at room temperature. Enriched mononuclear cells were washed with PBS twice and centrifuged at 500 × g for 10 min and measured the cell numbers and viability.

### Single‐Cell RNA and TCR Sequencing

Single‐cell libraries were prepared from freshly isolated PBMCs by using 10Xgenomics DNA V3 Reagent Kits. The cells and kit reagents were mixed with gel beads containing barcoded oligonucleotides (UMIs) and oligo dTs to form reaction vesicles called gel bead‐in‐emulsions (GEMs). Barcoded, full‐length cDNA is amplified via PCR with primers against common 5’ and 3’ ends added during GEM‐RT. This amplification reaction generates sufficient material to construct multiple libraries from the same sample, including both 5’ gene expression and T cell enriched libraries. The Single Cell 3’ Protocol produces Illumina‐ready sequencing libraries. The Single Cell V(D)J Reagent Kit protocol produces V(D)J enriched and 5’ gene expression Illumina‐ready sequencing libraries. Libraries were sequenced on the Illumina HiSeq 4000 platform under an effective concentration by Gene Denovo Biotechnology Co. (Guangzhou, China).

### Antibodies and Flow Cytometric Analysis

Morning blood samples were isolated by density centrifugation using Ficoll‐Paque PLUS. Post centrifugation, the mononuclear cell layer containing PBMCs was collected and added to a new universal tube, and the cells were washed twice with MACS buffer. For phenotypic characterization of T cells, isolated PBMCs were stained with a combination of fluorochrome‐conjugated antibodies (Table S1, Supporting Information). Data were acquired using a BD LSRFortessa Flow Cytometer. Data analysis and plotting were performed using FlowJo v9.

### Statistical Analysis—Single‐Cell Data Analysis

Raw gene expression matrices were generated for each sample by the Cell Ranger Pipeline (https://github.com/10XGenomics/cellranger) coupled with human reference version GRCh38. The output filtered gene expression matrices were analyzed by R software with the Seurat package.^[^ ^39^ ^]^ The first step was quality control, which filters cells with gene expression, unique UMI, and percent of the mitochondrial genes. To remove ambient contamination, the data for each sample using the R package sctransform was transformed and normalized. Furthermore, DoubletFinder was used to remove doublets in each sample. Principal component analysis showed there was batch effect among samples (Figure S7A, Supporting Information). The Harmony algorithm can accurately integrate single‐cell data from different technology platforms and batches.^[^ ^40^ ^]^ Thus, data from different samples using the Harmony algorithm (Figure S7B, Supporting Information) was integrated. Then, principal component analysis (PCA), tSNE, and UMAP clustering algorithms were used to visualize clustered cells in 2D space. Differentially expressed genes were identified using the FindAllMarkers function, and the top 5 genes were visualized in a heatmap using the DoHeatmap function. Immune cell subsets were identified based on the expression of marker genes. The Wilcox rank sum test was applied to compare T cell subtype percentages among samples in CEN, CO, and Control groups. Gene enrichment analysis was performed by the R package clusterProfile.^[^ ^41^ ^]^ TF analysis was performed by SCENIC (SCENIC: single‐cell regulatory network inference and clustering). TF‐gene regulation network analysis was performed using Cytoscape.^[^ ^42^ ^]^ Besides, DEsingle was used to identify differentially expressed genes between CEN versus Control and CO versus Control. Additionally, scRNA‐seq data of the PBMC from young and aged adults were collected from the Genome Sequence Archive in BIG Data Center, Beijing Institute of Genomics (BIG, https://bigd.big.ac.cn/gsa‐human/) with Project Accession No. PRJCA002865 and GSA Accession No. HSA000203;^[^ ^15^ ^]^ and immune cell annotation data from Mogilenko et al. were downloaded from https://artyomovlab.wustl.edu/immune‐aging/explore.html.^[^ ^16^ ^]^ The cohort published by Zheng et al. were composed of PBMC samples from 16 healthy nonfrail donors, which were recruited in the Zhongshan Ophthalmic Center. Among the above 16 individuals, there were 8 young adults (20–45 years old) and 8 old adults (60–80 years old).^[^ ^15^ ^]^ The cohort published by Mogilenko et al. consisted PBMC samples from 21 healthy, Caucasian, nonobese (BMI under 30) donors from Washington.^[^ ^16^ ^]^ Young donors (25–30 years old, BMI 21.2–28.9 kg m^−2^, *n* = 11) and old nonfrail donors (BMI 17.8–29.8 kg m^−2^, 62–70 years old, *n* = 10) were included.

### Aging Score Calculation

A total of 451 aging‐related genes of 37 gene sets were collected from Aging Atlas (https://ngdc.cncb.ac.cn/aging/index) (Table S2, Supporting Information).^[^ ^21^ ^]^ Subsequently, aging scores were calculated using the AddModuleScore function of Seurat package by taking the aging gene sets as features.

### Single‐Cell TCR Analysis

Cell Ranger was used for the TCR/BCR sequencing data processing. Cell Ranger aligns reads to all the V(D)J gene segments included in the reference. Cell barcodes are grouped into clonotypes, if they share the same set of productive CDR3 nucleotide sequences by exact match. The R package “immunarch” was used to analyze the characteristic of TCR. Also, STARTRAC^[^ ^43^ ^]^ was used to investigate the distribution and clonal expansion of TCR.

### Ethical Statement

The Ethics Committee of the Affiliated Hospital of Nantong University approved the study (approval number 2019‐K045).

### Patient Consent Statement

Written informed consent was received prior to participation of all samples.

## Conflict of Interest

The authors declare no conflict of interest.

## Author Contributions

C.D., Y.‐r.M., R.Z., and M.Y. contributed equally to this work. Conceptualization: C.D., X.Z., J.G., and Z.G. Data curation: C.D., R.Z., and T.L. Formal analysis: Y.M., M.Y., A.G., and Q.Z. Funding acquisition: Z.X., C.S., and Z.G. Investigation: C.D., R.Z., T.L., Y.B., C.S., Y.Y., Y.J., R.L., M.X., J.G., Z.Z., W.Z., M.H., and D.W. Methodology: C.D., Y.M., R.Z., A.G., X.Z., J.G., and Z.G. Project administration: C.D., R.Z., and Z.G. Resources: J.G. and Z.G. Software: Y.M. and X.G. Supervision: C.D., A.G., Z.X., C.S., J.G., and Z.G. Validation: C.S., X.Z., and Z.G. Visualization: Y.M., A.G., and X.G. Writing—original draft: C.D., Y.M., R.Z., and M.Y. Writing—review & editing: C.D., Z.X., X.Z., and Z.G.

## Supporting information

Supporting Information

Supplemental Table 1

Supplemental Table 2

## Data Availability

All data needed to evaluate the conclusions in the paper are present in the Supplementary Materials and/or avaible from corresponding author. The raw sequence data have been deposited in Genome Sequence Archive in BIG Data Center, Beijing Institute of Genomics (BIG, http://bigd.big.ac.cn/gsa‐human/) with Project Accession No.PRJCA011218 and GSA Accession No. HRA002867.
