# Supplementary material for: Single‐Cell Transcriptomics Reveals Longevity Immune Remodeling Features Shared by Centenarians and Their Offspring
Source: Adv Sci (Weinh). 2022 Nov 10;9(36):2204849. doi: 10.1002/advs.202204849 (PMC9799020; doi:10.1002/advs.202204849)
Supplement: Supplementary file 1 — Supporting Information [file ADVS-9-2204849-s001.pdf]

ADVANCED SCIENCE
Open Access

# Supporting Information

for *Adv. Sci.*, DOI 10.1002/advs.202204849

Single-Cell Transcriptomics Reveals Longevity Immune Remodeling Features Shared by Centenarians and Their Offspring

*Chen Dong, Ya-ru Miao, Rui Zhao, Mei Yang, An-yuan Guo, Zhong-hui Xue, Teng Li, Qiong Zhang, Yanfeng Bao, Chen Shen, Chi Sun, Ying Yang, Xi-xi Gu, Yi Jin, Rong Li, Min Xu, Jia-xin Guo, Zhi-ying Zong, Wei Zhou, Mei He, Dan-ni Wang, Jian-you Su, Xiao-ming Zhang, Xu-hui Zeng*, Jian-lin Gao* and Zhi-feng Gu**

Supporting Information

# Single-cell Transcriptomics Reveals Longevity Immune Remodeling Features Shared by Centenarians and Their Offspring

*Chen Dong†, Ya-ru Miao†, Rui Zhao†, Mei Yang†, An-yuan Guo, Zhong-hui Xue, Teng Li, Qiong Zhang, Yanfeng Bao, Chen Shen, Chi Sun, Ying Yang, Xi-xi Gu, Yi Jin, Rong Li, Min Xu, Jia-xin Guo, Zhi-ying Zong, Wei Zhou, Mei He, Dan-ni Wang, Jian-you Su, Xiao-ming Zhang, Xu-hui Zeng*, Jian-lin Gao*, Zhi-feng Gu**

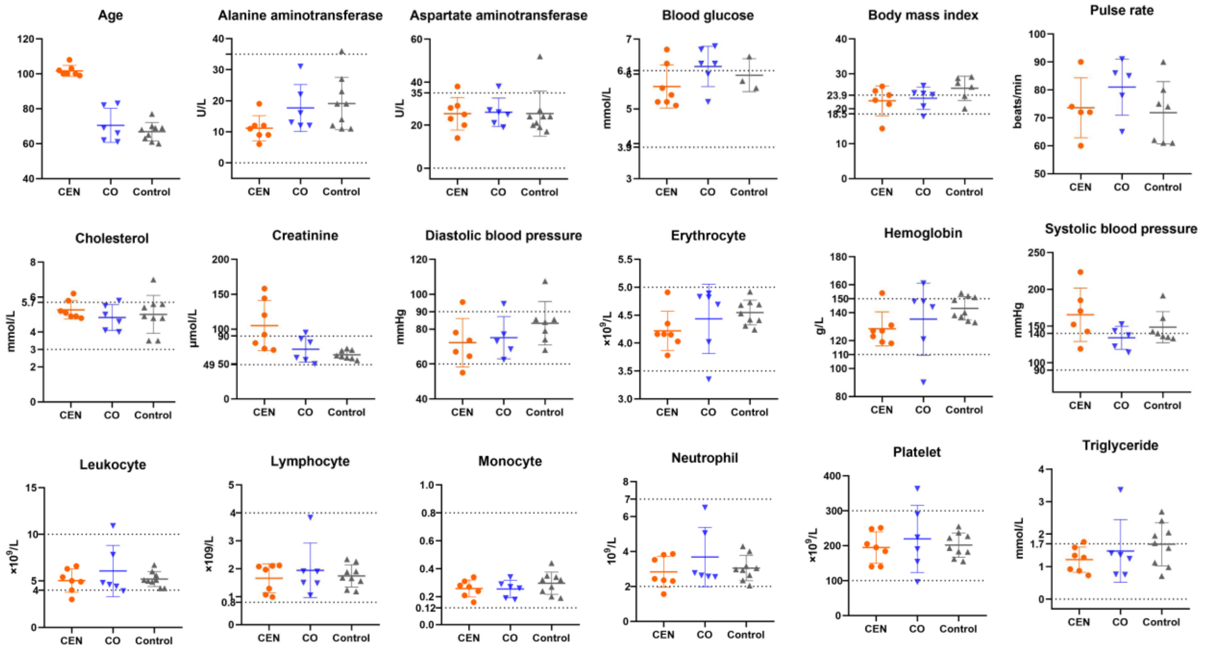


**Figure S1.** Bar plot of physiological indicators of CEN, CO, and Control samples. Dotted line indicates the normal range.

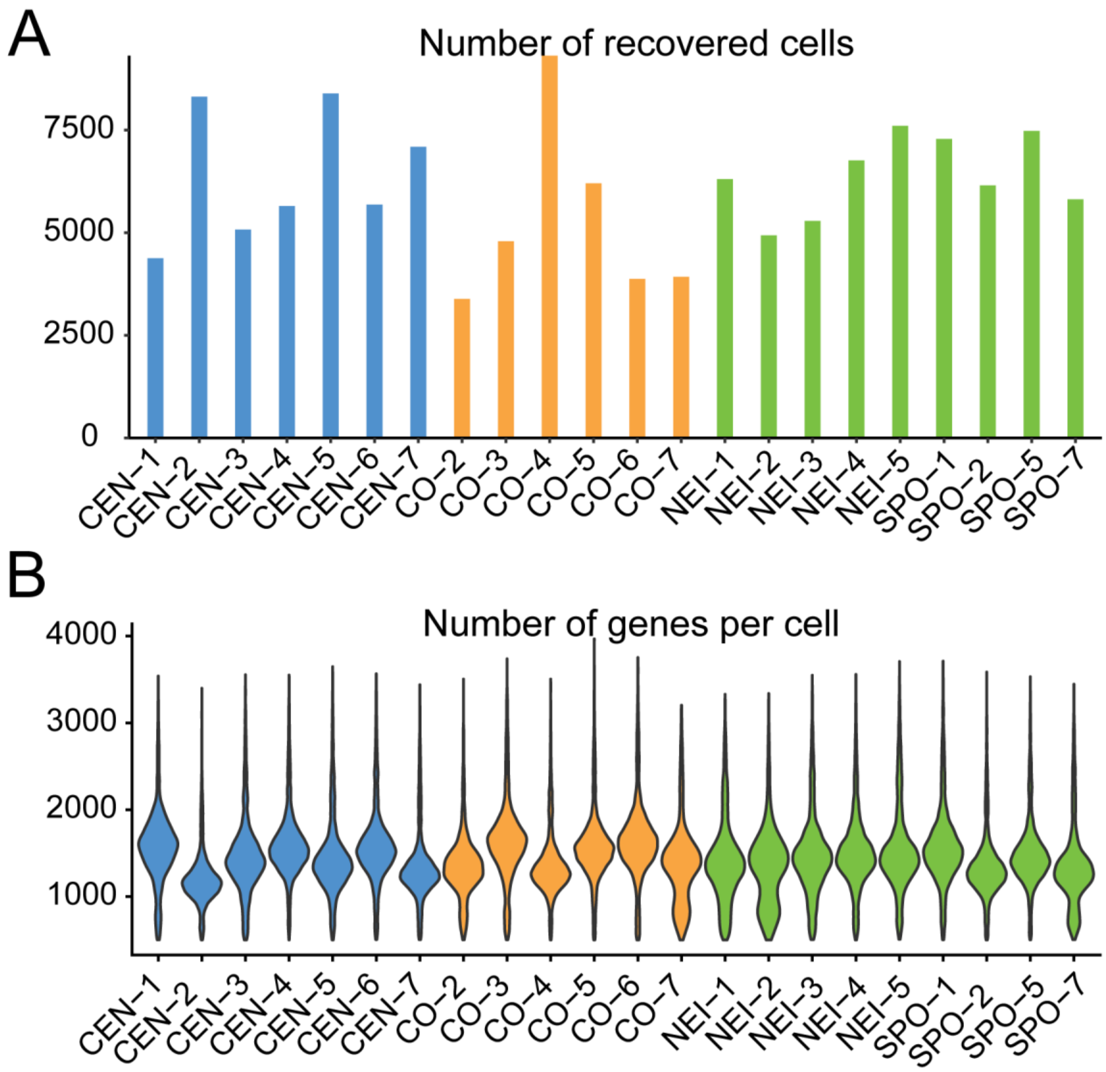


**Figure S2. A.** The number of recovered cells that passed quality control. **B.** the number of genes per cell in samples of CEN, CO, and Control samples.

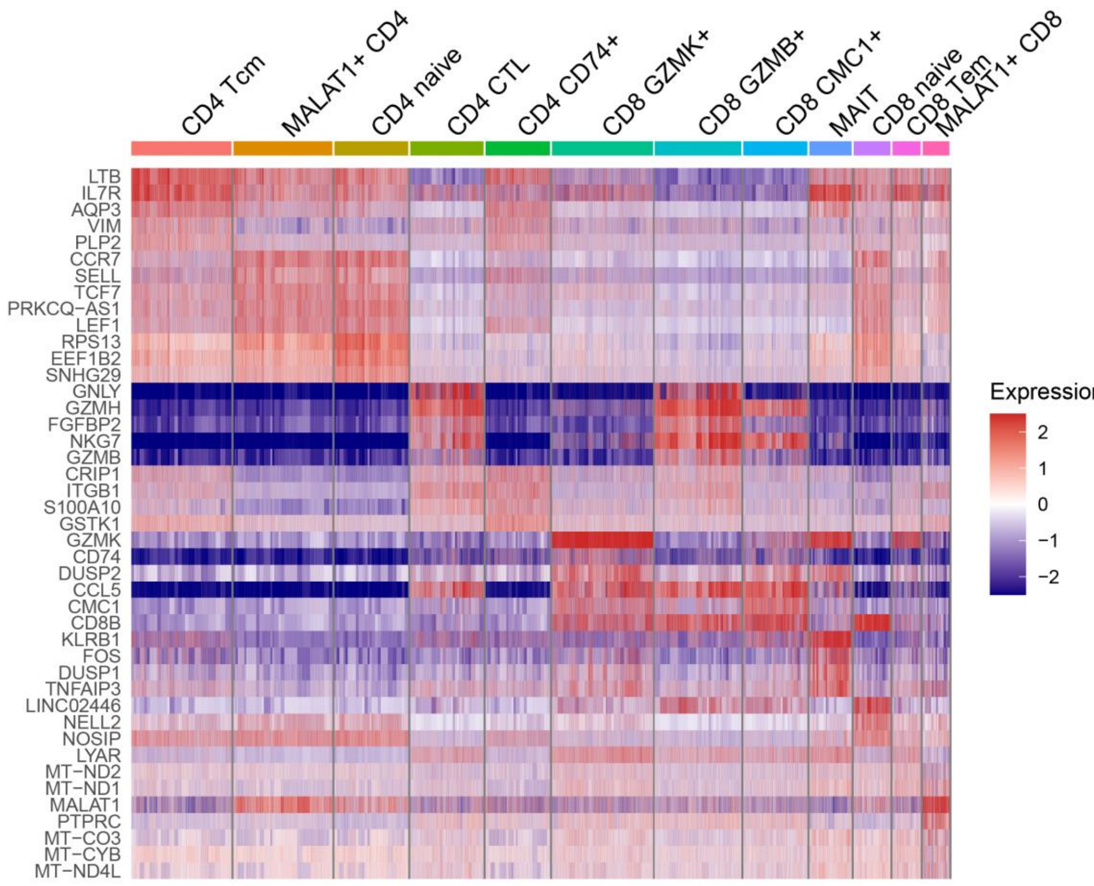


**Figure S3.** Heatmap of top5 DEGs of T cell clusters.

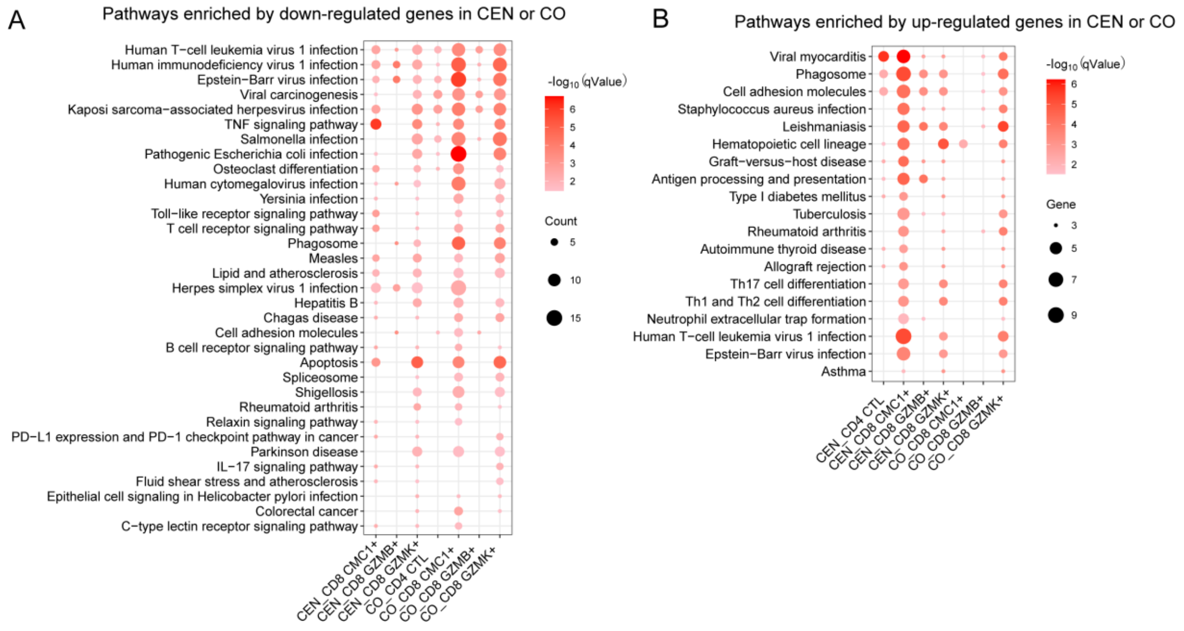

**Figure S4.** KEGG pathways enrichment by down-regulated (A) or up-regulated genes (B) in CPC cell clusters of CEN and CO, respectively.

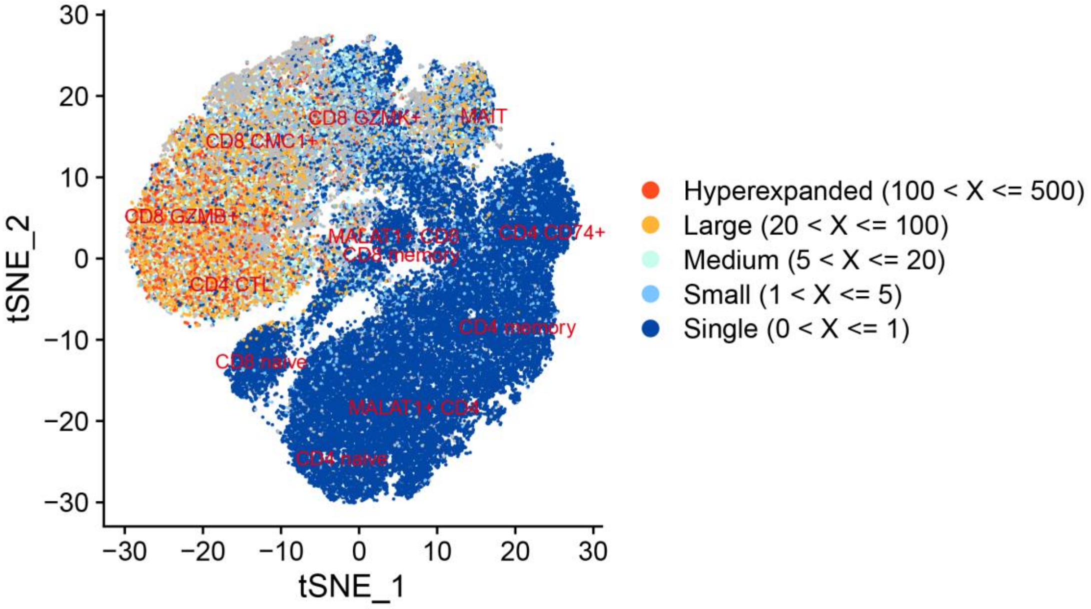


**Figure S5**. Two-dimensional tSNE visualization showing the distribution of T cell clone type frequency in T cell clusters.

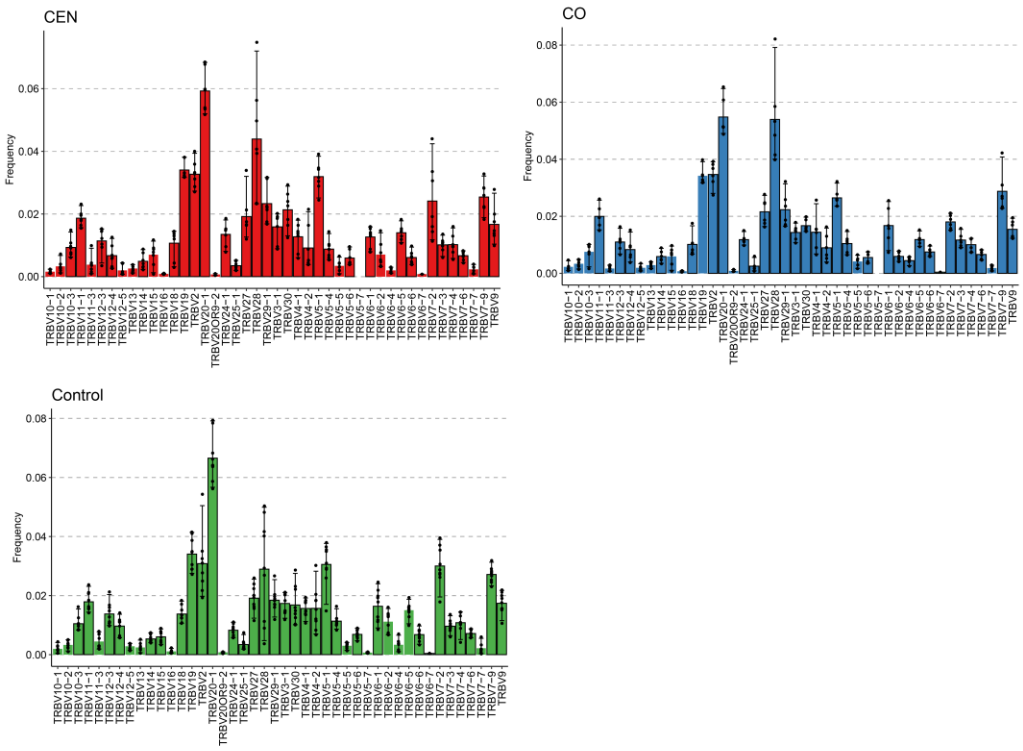


**Figure S6.** Barplot showing TRB v gene frequency in CEN, CO, and Control samples.

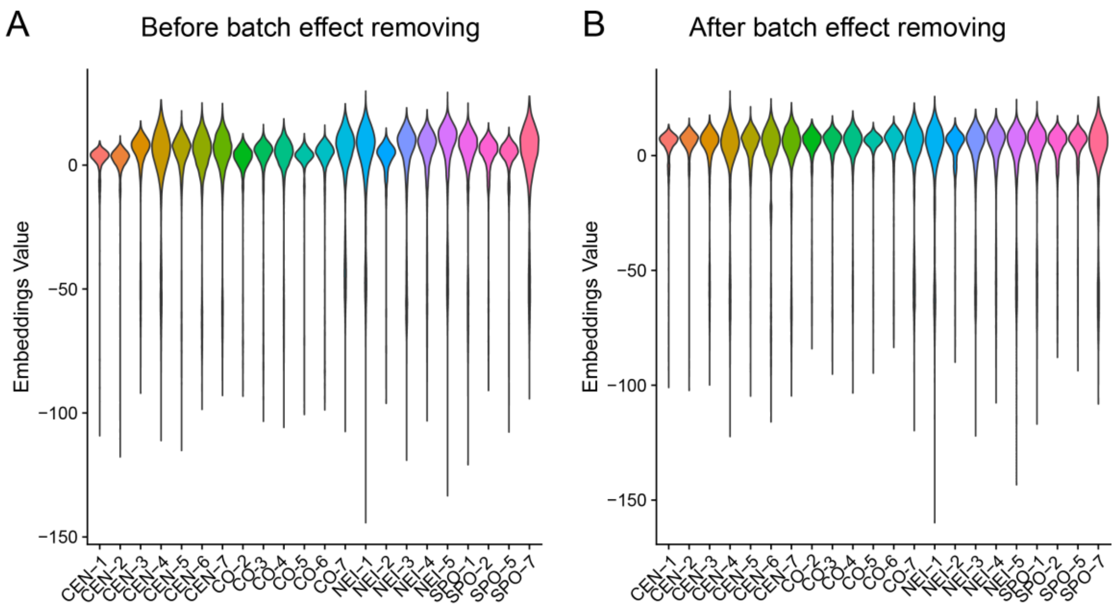


**Figure S7.** Violin plot shows the result of principal component analysis of each sample before (A) and after (B) batch effect removing.
