# Supplementary material for: Single‐Cell Transcriptomics Reveals Longevity Immune Remodeling Features Shared by Centenarians and Their Offspring
Source: Adv Sci (Weinh). 2022 Nov 10;9(36):2204849. doi: 10.1002/advs.202204849 (PMC9799020; doi:10.1002/advs.202204849)
Supplement: Supplementary file 2 — Supplemental Table 1 [file ADVS-9-2204849-s003.pdf]

ADVANCED SCIENCE
Open Access

Supporting Information

for *Adv. Sci.*, DOI 10.1002/advs.202204849

Single-Cell Transcriptomics Reveals Longevity Immune Remodeling Features Shared by Centenarians and Their Offspring

Chen Dong, Ya-ru Miao, Rui Zhao, Mei Yang, An-yuan Guo, Zhong-hui Xue, Teng Li, Qiong Zhang, Yanfeng Bao, Chen Shen, Chi Sun, Ying Yang, Xi-xi Gu, Yi Jin, Rong Li, Min Xu, Jia-xin Guo, Zhi-ying Zong, Wei Zhou, Mei He, Dan-ni Wang, Jian-you Su, Xiao-ming Zhang, Xu-hui Zeng, Jian-lin Gao* and Zhi-feng Gu**

Table S1. Key details of antibody in this study.

Antibody	Source	IDENTIFIER	Cat	Company
CD3	human	BUV395	564001	BD
CD4	human	BV510	566479	BD
CD8	human	A700	300920	Biolegend
FVS780		APCcy7		
GZMA	Human	PB	507207	Biolegend
GZMB	Human	PE	561142	BD
GZMK	Human	AF647	566655	BD
CD45RA	Human	BV711	304138	Biolegend
CCR7	Human	PC594	353236	Biolegend
